# Electrospun Membranes Anchored with g-C_3_N_4_/MoS_2_ for Highly Efficient Photocatalytic Degradation of Aflatoxin B_1_ under Visible Light

**DOI:** 10.3390/toxins15020133

**Published:** 2023-02-06

**Authors:** Ruixin Song, Liangtao Yao, Changpo Sun, Dechao Yu, Hui Lin, Guisheng Li, Zichao Lian, Songlin Zhuang, Dawei Zhang

**Affiliations:** 1 Engineering Research Center of Optical Instrument and System, Ministry of Education and Shanghai Key Lab of Modern Optical System, University of Shanghai for Science and Technology, No. 516 Jungong Road, Shanghai 200093, China; 2 Standards and Quality Center of National Food and Strategic Reserves Administration, No. 25 Yuetan North Street, Xicheng District, Beijing 100834, China; 3 Department of Chemistry, College of Science, University of Shanghai for Science and Technology, No. 516 Jungong Road, Shanghai 200093, China; 4 Fujian Provincial Key Laboratory for Advanced Micro-Nano Photonics Technology and Devices, Research Center for Photonics Technology, Quanzhou Normal University, Quanzhou 362046, China

**Keywords:** electrospun photocatalytic membranes, aflatoxin B_1_, flexible, visible light, g-C_3_N_4_/MoS_2_

## Abstract

The degradation of aflatoxin (AF) is a topic that always exists along with the food and feed industry. Photocatalytic degradation as an advanced oxidation technology has many benefits, including complete inorganic degradation, no secondary contamination, ease of activity under moderate conditions, and low cost compared with traditional physical, chemical, and biological strategies. However, photocatalysts are usually dispersed during photocatalytic reactions, resulting in energy and time consumption in the separation process. There is even a potential secondary pollution problem from the perspective of food safety. In this regard, three electrospun membranes anchored with g-C_3_N_4_/MoS_2_ composites were prepared for highly efficient photocatalytic degradation of aflatoxin B_1_ (AFB_1_) under visible light. These photocatalytic membranes were characterized by XRD, SEM, TEM, FTIR, and XPS. The factors influencing the degradation efficiency of AFB_1_, including pH values and initial concentrations, were also probed. The three kinds of photocatalytic membranes all exhibited excellent ability to degrade AFB_1_. Among them, the photocatalytic degradation efficiency of the photocatalytic membranes prepared by the coaxial methods reached 96.8%. The experiment is with an initial concentration of 0.5 μg/mL (500 PPb) after 60 min under visible light irradiation. The mechanism of degradation of AFB_1_ was also proposed based on active species trapping experiments. Moreover, the prepared photocatalytic membranes exhibited excellent photocatalytic activity even after five-fold use in the degradation of AFB_1_. These studies showed that electrospun membranes anchored with g-C_3_N_4_/MoS_2_ composites have a high photocatalytic ability which is easily removed from the reacted medium for reuse. Thereby, our study offers a highly effective, economical, and green solution for AFB_1_ degradation in the foodstuff for practical application.

## 1. Introduction

Aflatoxin B_1_ (AFB_1_) is a highly toxic mycotoxin produced by aspergillus species as secondary metabolites under specific conditions [1,2,3]. It can contaminate food in a variety of ways and get into the human food chain directly or indirectly, threatening human health because of its genetic toxicity, carcinogenesis, embryonic toxicity, teratogenic, and immunotoxicity [4,5]. Studies have shown that a large amount of AFB_1_ consumed quickly can cause liver damage, such as acute hepatitis and liver tissue hemorrhage. Long-term intake of AFB_1_ can lead to chronic poisoning symptoms, such as liver fibrosis, poor growth, infertility, fetal malformation, etc. [6]. The International Agency for Research on Cancer (IARC) has listed AFB_1_ as a type I carcinogen [7,8,9,10]. In order to ensure human health and safety, the maximum allowable limits of AFB_1_ in various foods are determined. In the European Commission, the maximum allowable limit of AFB_1_ in edible oil, grain, and cereal products is 2 μg/kg. In China, the maximum allowable limit of AFB_1_ in peanut and corn oil is 20 μg/kg, while that in other vegetable oils is 10 μg/kg. In the United States, the maximum allowable limit of total aflatoxin (AFB_1_ + AFB_2_ + AFG_1_ + AFG_2_) in foods is 20 μg/kg. Meanwhile, animals fed with feed contaminated by AFB_1_ for a long time will increase the probability of disease and reduce feed conversion efficiency [11,12].

Various approaches have been reported for the detoxification of AFB_1_, including physical, chemical, and biological treatments. The most common physical detoxification method uses adsorbents in which AFB_1_ can be adsorbed during the process of detoxification [13,14]. Although many adsorbents, such as diatomite and montmorillonite, are used in practical applications, some common drawbacks include poor adsorptive efficiency, weak selectivity, high-cost recyclability, and even non-renewability. Chemical detoxification methods mainly use chlorine dioxide, ozone, sodium hypochlorite, and other chemicals to degrade AFB_1_ [15,16]. However, the problem of chemical residues has not been effectively solved and may cause secondary pollution. Another approach is to employ biodegradable enzymes or microorganisms to decompose AFB_1_ [17,18]. However, the application of the biological method is limited because the enzyme or bacteria agents are sensitive to environmental temperature, humidity, pH value, and the cost is high. Moreover, the increasing concern about food safety and the quality of the environment has prompted researchers to seek an efficient, safe, rigorous, and affordable technology to degrade AFB_1_.

Photocatalytic technology was developed in the 1970s [19] and is increasingly used in mycotoxins’ degradation [20,21,22]. In a photocatalytic reaction, when light with appropriate energy (hν ≥ E_g_) falls on photocatalytic materials, electrons (e^−^) get excited from the valence band (VB) to the conduction band (CB), leaving behind holes (h^+^). Then, these photogenerated charges (e^−^ and h^+^) migrate from the inside to the surface of the photocatalyst and interact with O_2_, H_2_O, or OH^−^ around to produce •O_2_^−^ and •OH with strong oxidation, which can degrade AFB_1_ and convert it into less hazardous compounds such as small organic acids, CO_2_, or H_2_O [23,24]. Compared with the physical, chemical, and biological treatments mentioned above, detoxifying mycotoxins using a photocatalytic approach is an emerging and promising strategy because of several advantages, including being free from secondary pollution, having mild conditions, and being economical, highly efficient, and environmental-friendly. Different studies have been carried out for detoxifying mycotoxin, including AFB_1_ and deoxynivalenol (DONs), using photocatalytic technology (Table 1). Recently, by using the experiments of isotope tracing, electron spin resonance, and active species trapping, Mao et al. found that preferentially inactivating the C8=C9 site by the addition reaction of hydroxyl radical was the main pathway for the detoxification of aflatoxin B1 [22]. Furthermore, hydroxyl radicals were most likely to react with the C9 site and then form AFB_1_-9-hydroxy through oxidative addition reaction, which was verified by theoretical calculations.

When the photocatalysts mentioned above were used to degrade AFB_1_ and DONs, the photocatalysts were generally suspended during the photocatalytic process [22,23,24,25,26,27,28,29]. As a result, the photocatalyst powders were easy to agglomerate and the separation process after the photocatalytic reaction required a lot of energy, which limited its large-scale application [31]. It is an attractive solution to prepare membranes by electrospinning as the carrier of photocatalysts. Electrospinning can produce fibers of tens to hundreds of nanometers in diameter with good mechanical properties, which can easily immobilize and recycle photocatalysts [32,33]. Thus, the energy consumption in the separation process and possible secondary pollution are reduced. Up to now, we have not found any reports on photocatalytic degradation of AFB_1_ using photocatalysts immobilized on electrospun membranes.

AFB_1_ is often produced during the storage, transportation, and production of foods or food ingredients [2,3]; so, the safety and stability of photocatalysts must be considered. Among the numerous photocatalysts, graphitic carbon nitride (g-C_3_N_4_) has gained the intensive attention of many researchers, as this metal-free polymeric n-type semiconductor is non-toxic, chemically stable, thermally stable, and easily modified [34]. However, the pristine g-C_3_N_4_ is usually restricted by unsatisfactory photocatalytic efficiency due to insufficient solar light absorption and the fast recombination of photogenerated electron–hole pairs [35]. In order to improve the photocatalytic efficiency of g-C_3_N_4_, it is a reasonable strategy to construct heterostructures with other narrow-band gap semiconductors to provide more active sites and inhibit the recombination of photogenerated charges. Molybdenum disulfide (MoS_2_) consists of three-dimensional stacked atomic layers with direct and indirect band gaps of 1.90 eV and 1.20 eV. It has become one of the most popular emerging co-catalysts due to its appropriate band structure, low cost, non-toxic, and exhibits excellent sunlight harvesting capability [36]. Therefore, it is a good idea to composite g-C_3_N_4_ with MoS_2_ to form effective heterostructures to enhance the visible light absorption and reduce the recombination of photogenerated electron–hole pairs owing to their matching band-edge positions for photocatalytic application [37]. To the best of our knowledge, the attempt to use electrospun membranes anchored with g-C_3_N_4_/MoS_2_ to degrade AFB_1_ under visible light irradiation has not been reported.

## 2. Results and Discussion

Based on the above considerations, we prepared g-C_3_N_4_/MoS_2_ composites by calcination and hydrothermal methods and investigated their photocatalytic properties. Then, the prepared photocatalysts were dispersed in the polymer electrospinning solution synthesized by polyacrylonitrile (PAN), and flexible electrospun membranes with different structures anchored with g-C_3_N_4_/MoS_2_ composites were prepared by uniaxial and coaxial methods, respectively. The as-prepared photocatalysts and flexible electrospun membranes (S_1_, S_2_, and S_3_) were characterized by scanning electron microscopy (SEM), transmission electron microscopy (TEM), X-ray diffraction (XRD), Fourier-transform infrared spectroscopy (FTIR), X-ray photoelectron spectroscopy (XPS), and diffuse reflectance spectra (DRS). The photocatalytic efficiency of electrospun membranes for degradation of AFB_1_ under visible light irradiation in an aqueous medium was investigated. Effects of factors such as pH value and the initial concentration of AFB_1_ were also studied. Active species trapping experiments analyzed the mechanism of photocatalytic degradation of AFB1. In addition, the effect of recycling on photocatalytic efficiency was also evaluated.

### 2.1. Characterization of the PAN-g-C_3_N_4_/MoS_2_ Electrospun Membranes

**Figure 1:**
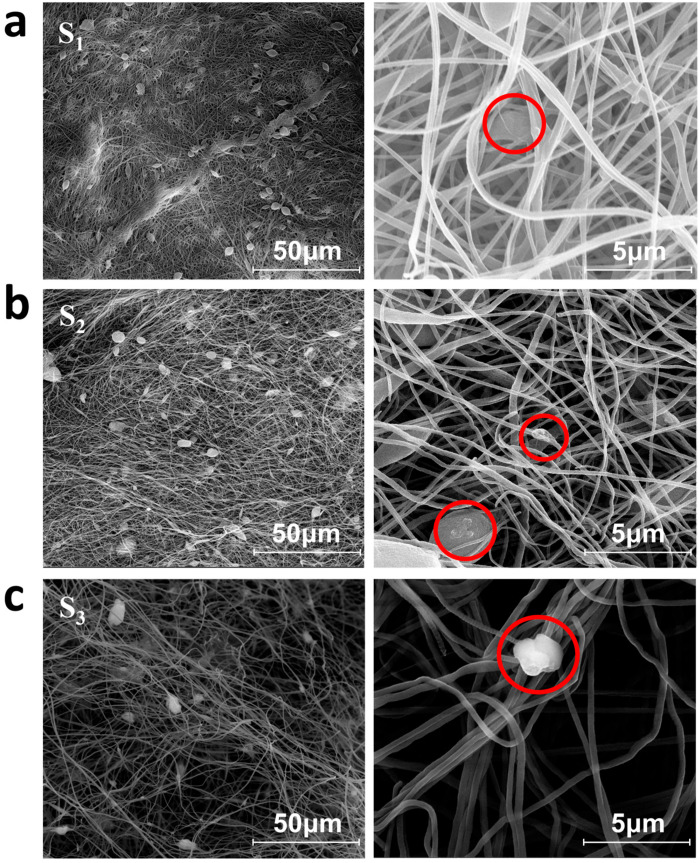
SEM images of electrospun membranes anchored with g-C_3_N_4_/MoS_2_ prepared by different processes: (**a**) S_1_~mostly wrapped, (**b**) S_2_~partially exposed, and (**c**) S_3_~fully exposed.

To study the morphologies of electrospun membranes anchored with g-C_3_N_4_/MoS_2_ prepared by different processes, S_1_, S_2_, and S_3_ were examined by SEM (Figure 1). It could be seen spindle-like beads wrapped with g-C_3_N_4_/MoS_2_ on S_1_ (Figure 1a), which indicated the photocatalysts were successfully immobilized on electrospun membranes. Many other researchers have prepared a series of photocatalytic membranes by similar methods [38]. However, most of the photocatalysts in this kind of membrane were wrapped by polymers, which hindered light absorption and was not conducive to the migration of photogenerated charges to the active sites. Therefore, polyethylene oxide (PEO) was added into the electrospinning solution, which is very soluble in water, and the obtained electrospun membranes were treated with an ultrasonic water bath to expose more photocatalysts. From the red circles marked (Figure 1b), it could be confirmed that pores formed by removing PEO after post-treatment, so that more photocatalysts were exposed and the photocatalytic efficiency was enhanced accordingly. To further expose the photocatalysts, coaxial electrospinning and ultrasonic water washing treatment were adopted to prepare S_3_. Compared with S_1_ and S_2_, the spindle-like beads were greatly reduced, and the photocatalysts that were completely exposed due to PEO could be obliterated. The way electrospun nanofibers bound the photocatalysts (Figure 1c) and wave-like folds caused by the removal of PEO could be observed in the bright area around the red circle. With the increase in photocatalysts exposure, it can be speculated that the photocatalytic efficiency should be improved correspondingly.

**Figure 2:**
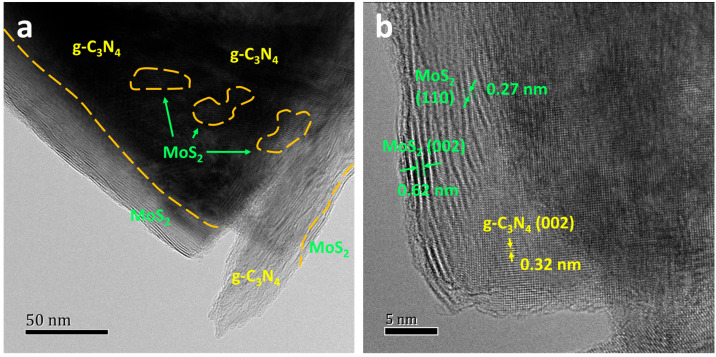
(**a**) TEM and (**b**) HRTEM images of g-C_3_N_4_/MoS_2_ composites.

The morphologies of the g-C_3_N_4_/MoS_2_ composites were further studied by TEM and HRTEM (Figure 2). It was observed that the well-crystallized MoS_2_ lines were loaded on g-C_3_N_4_ (Figure 2a). Furthermore, many clear lattice fringes were shown in the HRTEM image (Figure 2b), indicating that good crystallinity has been obtained. Three sets of different lattices were found with the d-spacing of 0.62 nm, 0.32 nm, and 0.27 nm, respectively, corresponding to the (002) plane of MoS_2_, the (002) plane of g-C_3_N_4_, and the (110) plane of MoS_2_, respectively [39]. Meanwhile, the interface between g-C_3_N_4_ and MoS_2_ could also be perceived, indicating that the heterostructures were successfully formed between g-C_3_N_4_ and MoS_2_.

The crystal structure and composition of g-C_3_N_4_/MoS_2_, S_1_, S_2_, and S_3_ were confirmed with X-ray diffraction (XRD). In addition, the XRD pattern of g-C_3_N_4_ and MoS_2_ was displayed to be compared with g-C_3_N_4_/MoS_2_ (Figure S1), which provided more detailed data. As shown in Figure S1a, several diffraction peaks could be observed at 2θ = 14.5°, 32.8°, 33.66°, 39.68°, 44.32°, and 49.92°, corresponding to (002), (100), (101), (103), (006), and (105) planes of MoS_2_ (JCPDS: 37-1492), respectively [40]. Compared with the standard card, the diffraction peaks of g-C_3_N_4_/MoS_2_ and MoS_2_ shifted slightly to a bigger angle, which might be due to the residual stress in the material [41]. As shown in Figure S1b, the diffraction peak of g-C_3_N_4_, which appeared at 2θ = 13.14°, was assigned to the (001) plane, attributed to the triazine unit, and the strong peak located at 28.02° was the typical (002) diffraction plane ascribed to the inter-planar stacking of the aromatic system in g-C_3_N_4_ (JCPDS: 87-1526) [29]. By contrast, the diffraction peak of g-C_3_N_4_/MoS_2_ shifted to a smaller angle, implying the interaction between the g-C_3_N_4_ and MoS_2_. Through the Scherrer formula (Supplementary Information), the crystallite size of g-C_3_N_4_/MoS_2_ at the (002) plane could be estimated to be 98 Å, more significant than the crystallite size of g-C_3_N_4_ at the (002) plane (88 Å), which might be attributed to the improvement of crystallinity after annealing.

**Figure 3:**
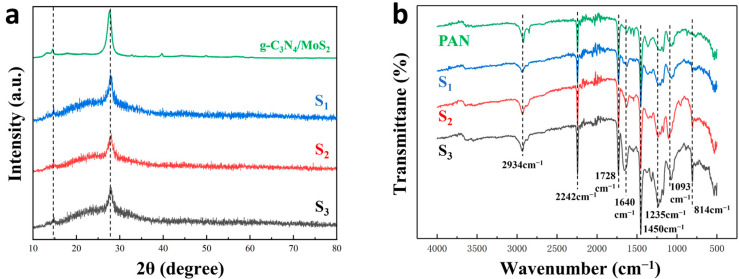
(**a**) XRD patterns and (**b**) FTIR spectra of S_1_, S_2_, and S_3_.

The XRD patterns of S_1_, S_2_, and S_3_ were generally very similar (Figure 3a) since they were all composed of PAN and g-C_3_N_4_/MoS_2_. The only difference lay in the spatial structure of the photocatalysts and PAN nanofibers. Obvious diffraction peaks belonging to MoS_2_ and g-C_3_N_4_ could be observed at 2θ = 14.72° and 27.5° in the XRD patterns of S_1_, S_2_, and S_3_, respectively. Additionally, wide bumps could be observed in the range of 15–30°, similar to the work of Xie et al. [42], representing the amorphous PAN macromolecules. The results of XRD patterns could confirm the successful combination of g-C_3_N_4_/MoS_2_ composites and PAN electrospun membranes. Other diffraction peaks of g-C_3_N_4_/MoS_2_ were not found in the XRD patterns of S_1_, S_2_, and S_3_ due to the low content of photocatalysts and the amorphous nature of PAN.

The FTIR spectra of the different electrospun membranes were measured (Figure 3b). For pure PAN electrospun membrane, the peaks at 2934 cm^−1^, 2242 cm^−1^, 1728 cm^−1^, 1450 cm^−1^, and 1093 cm^−1^ were assigned to the stretching vibration of methylene –CH_2_–, stretching vibration of C≡N, stretching vibration of C=O, bending vibration of –CH_2_–, and stretching vibration of the C–N bonds [42,43,44]. Compared with pure PAN electrospun membrane, the C–N stretching vibration absorption peak of g-C_3_N_4_ located at 1235 cm^−1^ and 1640 cm^−1^, and the characteristic peak of the 3-s-triazine structure located at 814 cm^−1^, appeared in the FTIR spectra of S_1_, S_2_, and S_3_ [45,46]. Therefore, the FTIR results further demonstrated the successful loading of photocatalysts on electrospun membranes. However, due to the low content of MoS_2_, its characteristic peaks failed to be observed. It should be noted that the intensity and area of the peaks assigned to g-C_3_N_4_ increased in turn from S_1_ to S_3_, indicating more photocatalysts were exposed, which was beneficial to improve photocatalytic efficiency.

**Figure 4:**
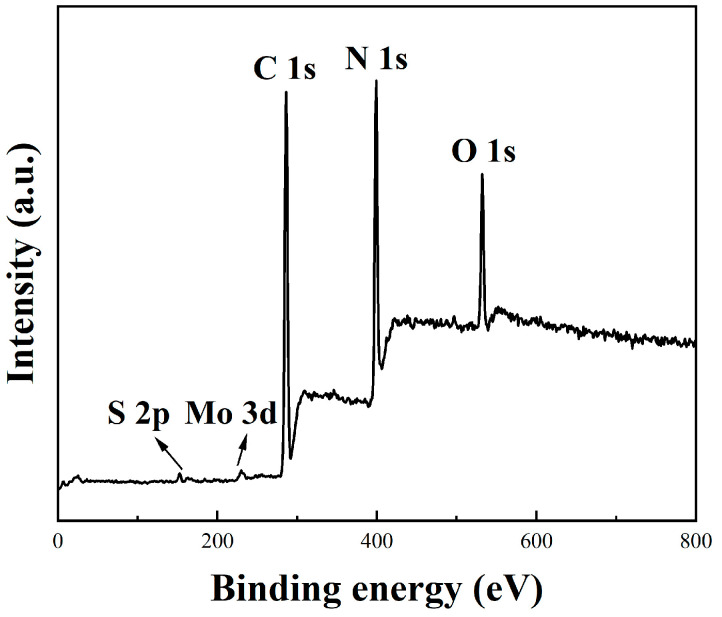
The full-scale XPS survey spectra of S_3_.

**Figure 5:**
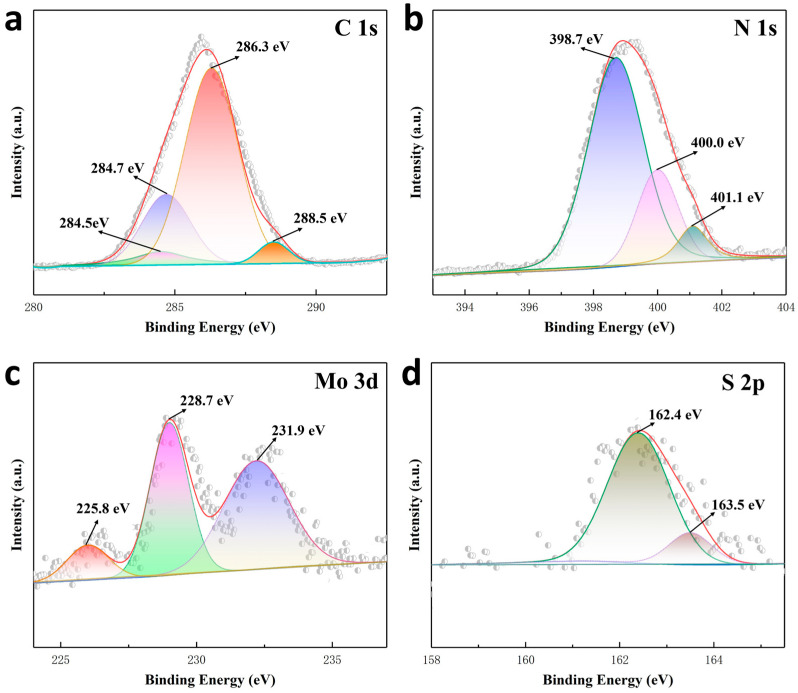
The high-resolution XPS spectra of S_3_: (**a**) C 1s, (**b**) N 1s, (**c**) Mo 3d, and (**d**) S 2p.

The chemical status and bonding structures of the PAN-g-C_3_N_4_/MoS_2_ electrospun membranes were analyzed by X-ray photoelectron spectroscopy (XPS). The full-scale XPS survey spectra revealed the existence of C, N, Mo, and S elements (Figure 4). In addition, the peak differentiation imitating the four elements was studied to further understand the detailed composition (Figure 5). The XPS spectra of C 1s could be deconvoluted into four peaks (Figure 5a), wherein the peaks at 284.5 eV and 286.3 eV were attributed to the sp^2^ C–C bonds and C-NH_2_ species of the g-C_3_N_4_ [33]. The peak at 284.7 eV (sp^2^ C-C) belonged to C 1s of PAN, and the peak at 288.5 eV could be attributed to the carbon in N-C=N [47]. The XPS spectra of N 1 s had three peaks at 398.7 eV, 400.0 eV, and 401.1 eV, respectively (Figure 5b), which could be attributed to the sp^2^ hybridized nitrogen in C-N=C, tertiary nitrogen N-(C)_3_ groups, and free amino groups (C-N-H) [33]. Three peaks in the high-resolution XPS spectra of Mo 3d at 225.8 eV, 228.7 eV, and 231.9 eV were further revealed (Figure 5c), belonging to S 2s, Mo 3d_5/2_, and Mo 3d_3/2_, respectively [47]. It could be confirmed that the Mo element in g-C_3_N_4_/MoS_2_ was mainly presented in the state of Mo^4+^. Regarding the XPS spectra of S 2p (Figure 5d), two major peaks at 162.4 eV and 163.5 eV could be attributed to S 2p_3/2_ and S 2p_1/2_, respectively [47]. The XPS results verified that the g-C_3_N_4_/MoS_2_ was successfully anchored with electrospun PAN membranes.

**Figure 6:**
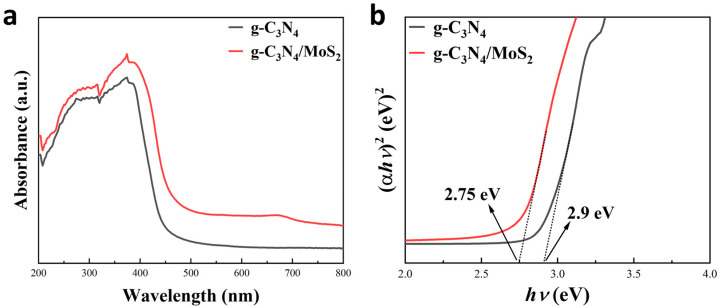
(**a**) Diffuse reflectance spectra (DRS) of g-C_3_N_4_ and g-C_3_N_4_/MoS_2_; (**b**) band gaps estimated respectively by the Kubelka–Munk equation from DRS data.

Figure 6a illustrates the DRS spectra of g-C_3_N_4_ and g-C_3_N_4_/MoS_2_ powders. Compared with pure g-C_3_N_4_, the absorption of g-C_3_N_4_/MoS_2_ has stronger intensity at the UV-visible light range and an obvious red-shift, which meant that the compounding of MoS_2_ effectively broadens and strengthens the light absorption. The heterojunction constructed between g-C_3_N_4_ and MoS_2_ changes the optical properties of hybrid materials, promoting the light absorption, and could improve the photocatalytic activity under visible-light irradiation.

The results of UV-Vis DRS were used to calculate the band gap energy (*E_g_*) of the material through the Kubelka–Munk formula (1):

*αhν* = *C*(*hv*−*E*_g_)*^n^*^/2^ (1)

where *α*, *h*, *ν*, and *C* are the absorption coefficient, Planck constant, optical frequency, and constant, respectively. The value of *n* is determined by the material properties. Through the Kubelka–Munk formula, the integral band gap of g-C_3_N_4_/MoS_2_ could be estimated to be 2.75 eV, while that of g-C_3_N_4_ was approximated to be 2.9 eV (Figure 6b). Moreover, g-C_3_N_4_/MoS_2_ with a narrower band gap should have better photocatalytic performance, according to a previous study [48].

**Figure 7:**
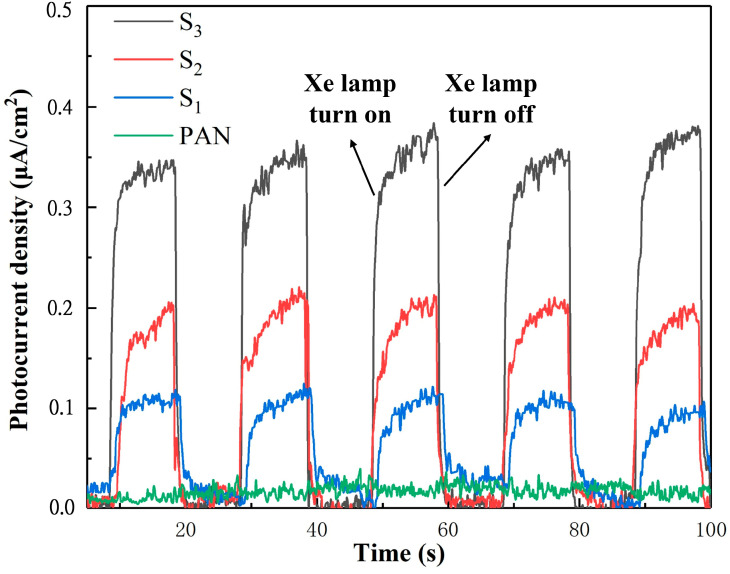
Transient photocurrent response curves of S_1_, S_2_, S_3_, and PAN electrospun membrane.

Furthermore, the transient photocurrent (TPC) response of the as-prepared S_1_, S_2_, S_3_, and PAN electrospun membrane was displayed (Figure 7) under the condition of light on and off illuminating by a visible light source (Xe lamp, λ ≥ 420 nm). It is known that the higher the photocurrent intensity, the higher the separation rate of photogenerated carriers. Obviously, PAN electrospun membrane had no response to visible light radiation, whereas the photocurrent density of S_1_, S_2_, and S_3_ significantly increased in turn when the Xe lamp was turned on, indicating that more photogenerated charges were generated, which was mainly due to the increasingly exposed g-C_3_N_4_/MoS_2_ from S_1_ to S_3_. Therefore, the photocatalysts could be completely exposed by optimizing the preparation method to not only enhance the harvest of light but also promote the transfer of photogenerated charges from the inner to the surface, which might improve the photocatalytic efficiency effectively.

### 2.2. Photocatalysis and Recycling Performance

**Figure 8:**
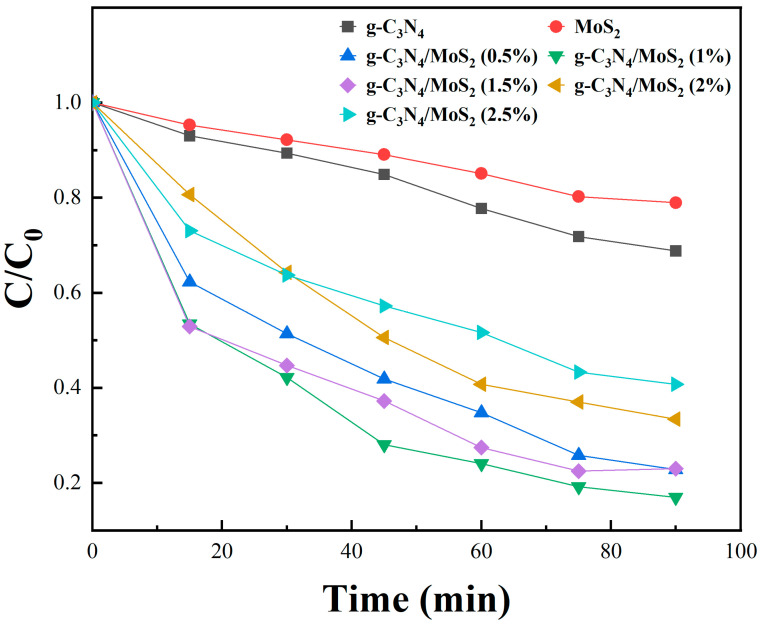
Photocatalytic degradation of RhB over g-C_3_N_4_/MoS_2_ with different weight ratios of MoS_2_.

Figure 8 shows the photocatalytic degradation of RhB (10 mg/mL) over g-C_3_N_4_/MoS_2_ with different mass ratios of MoS_2_ under visible light irradiation. It can be seen that g-C_3_N_4_/MoS_2_ (1%) had the highest photocatalytic activity, the degradation rate of RhB over which was close to 85% after 90 min. On the other hand, the degradation rate of g-C_3_N_4_ and MoS_2_ to RhB was about 32% and 20%, respectively, obviously inefficient in comparison with that of the composite photocatalyst. These results confirmed that the strategy of small amount of compounding MoS_2_ with g-C_3_N_4_ was workable to promote photocatalytic activity, and the best mass ratio of MoS_2_ in g-C_3_N_4_/MoS_2_ is 1%.

**Figure 9:**
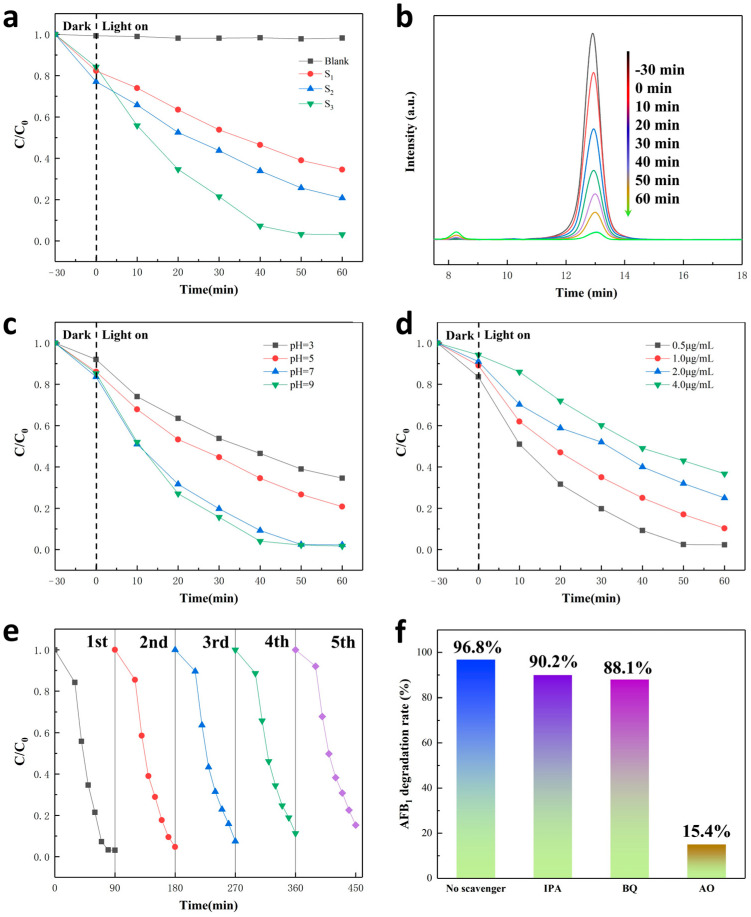
(**a**) Photocatalytic degradation efficiencies of AFB_1_ with as-prepared S_1_, S_2_, and S_3_ under visible light irradiation. (**b**) HPLC chromatogram of AFB_1_ photocatalytic degradation with S_3_ under visible light irradiation at different times. (**c**) The photocatalytic activity of S_3_ for degradation of AFB_1_ at different pH values. (**d**) The photocatalytic activity of S_3_ for degradation of AFB_1_ with different initial concentrations. (**e**) The photocatalytic activity of S_3_ for degradation of AFB_1_ for five cycles. (**f**) Photocatalytic activities of S_3_ for the degradation of AFB_1_ in the presence of different scavengers.

The photocatalytic performances were comparatively evaluated by photocatalytic degradation of AFB_1_ aqueous solution under visible light irradiation, and AFB_1_ aqueous solution without photocatalytic membrane was used as the control group (Figure 9). Before photocatalytic degradation under visible light irradiation, the AFB_1_ aqueous solution immersed with S_1_, S_2_, and S_3_ was kept in darkness for 30 min to achieve adsorption/desorption equilibrium, and the duration of photocatalytic reaction was 60 min.

It could be observed that for the blank experiment without a photocatalytic membrane, the concentration of AFB_1_ was unchanged under visible light irradiation. The photocatalytic activity of S_1_, S_2_, and S_3_ was significantly improved, and the photodegradation efficiency was up to 65.5%, 79.2%, and 96.8% in 60 min, respectively (Figure 9a). These results showed that the degradation of AFB_1_ was mainly due to a photocatalytic reaction. As we speculated, the efficiency of photocatalytic degradation of AFB_1_ by S_1_, S_2_, and S_3_ increased in turn. S_3_ showed greatly higher photocatalytic efficiency with a degradation rate of 31.3% and 17.6% higher than S_1_ and S_2_, respectively. This implied that g-C_3_N_4_/MoS_2_ anchored on electrospun PAN membranes played an important role in the photocatalytic activity of AFB_1_ degradation. As the g-C_3_N_4_/MoS_2_ anchored on S_3_ were utterly exposed, the light-harvesting ability was enhanced compared with S_1_ and S_2_. Thus, many photogenerated charges were produced in g-C_3_N_4_/MoS_2_ and more easily transferred to the surface of the photocatalyst because they were not wrapped by the polymer. More importantly, this fully exposed g-C_3_N_4_/MoS_2_ provided more active sites and greatly enhanced the photocatalytic efficiency. The high-performance liquid chromatography (HPLC) chromatogram of AFB_1_ aqueous solution concentrations with the irradiation time was also demonstrated (Figure 9b).

In a typical photocatalytic process, many factors affect photocatalytic performance. Besides the basic properties (crystal structure, particle size, specific surface area, and surface hydroxyl group) and carrier of the photocatalysts, external environmental factors such as light source, irradiation time, temperature, pH value, and initial concentration of reactants also make a certain sense [49]. In this study, the influence of pH values and initial concentrations of AFB_1_ on photocatalytic efficiency was estimated, which were two variable factors in practical application.

S_3_ was used to study the photocatalytic efficiency at pH values of 3, 5, 7, and 9, whereas the concentrations of AFB_1_ were kept constant (Figure 9c). It was observed that the degradation of AFB_1_ was suppressed in an acidic aqueous solution. With the increase in pH value, the photocatalytic degradation rates of AFB_1_ increased accordingly. In the neutral solution with a pH value of 7, nearly 17% of AFB_1_ was adsorbed after 30 min. However, in the acidic solution with pH values of 3 and 5, only 8% and 13% of AFB_1_ were adsorbed, indicating that the high photocatalytic degradation efficiency might come from high adsorption. The photocatalytic membranes and AFB1 (pH = 5) were positively charged in an acidic solution [26]. The absorption of AFB1 on the active site was low due to the repulsive force between the photocatalytic membranes and AFB1 [26,38]. Subsequently, the photocatalytic efficiency was weakened.

For the same reason, in an alkaline solution with a pH value of 9, there was a similar repulsive force between the photocatalytic membranes and AFB_1_. However, the photocatalytic degradation efficiency was not decreased but instead slightly increased. The reason might be that AFB_1_ was unstable in the alkaline environment [50]. To investigate the effect of the AFB_1_ initial concentration on the photocatalytic degradation efficiency, S_3_ was soaked in different initial concentrations of AFB_1_ (0.5–4 μg/mL, i.e., 500–4000 PPb) with a pH value of 7 (Figure 9d). It was observed that the photocatalytic degradation efficiency was inversely related to AFB_1_ initial concentration. The AFB_1_ degradation efficiencies were 97.5% and 63.3% at initial concentrations of 500 and 4000 PPb, respectively. This could be assigned to a constant number of active sites on the photocatalytic membrane. With the increase of initial concentrations and the proceeding of the photocatalytic reaction, competitive adsorption of AFB_1_ and its intermediates on the photocatalytic membranes would be aggravated, subsequently affecting the harvest of light and forming a barrier against photoexcitation in g-C_3_N_4_/MoS_2_ [28,51].

For the practical application of the photocatalytic membranes, five consecutive photocatalytic experiments were carried out using S_3_ under the same experimental conditions with proper washing and drying after each cycle (Figure 9e). The reproducibility results of AFB_1_ degradation by S_3_ showed that although the degradation pace decreased slightly after each photocatalytic degradation test, the degradation rate reached more than 85% overall. The slight decrease in degradation rate might be due to the contaminant of reused samples during the recovery step by the intermediate products produced in the photocatalytic degradation of AFB_1_. The recyclability of the photocatalytic membranes verified the possibility of practical application and a better economic benefit.

To better understand the mechanism of photocatalytic degradation of AFB_1_ by the PAN-g-C_3_N_4_/MoS_2_ electrospun membranes, the active species trapping experiments were carried out using S_3_ under the same conditions described above (Figure 9f). Isopropanol (IPA), 1,4-benzoquinone (BQ), and ammonium oxalate (AO) were employed as the scavengers for hydroxyl radicals (•OH), super-oxide anion radicals (•O_2_^−^), and photogenerated holes (h^+^), respectively [52]. After 60 min of visible light irradiation, the degradation rate of AFB_1_ without a sacrificial agent was 96.8%, and for others with scavengers IPA, BQ, and AO, the degradation rate was 90.2%, 88.1%, and 15.4%, respectively. Therefore, it could be confirmed that h^+^ was the main active specie in the reaction process.

### 2.3. Mechanism for Enhanced Degradation Performance

**Figure 10:**
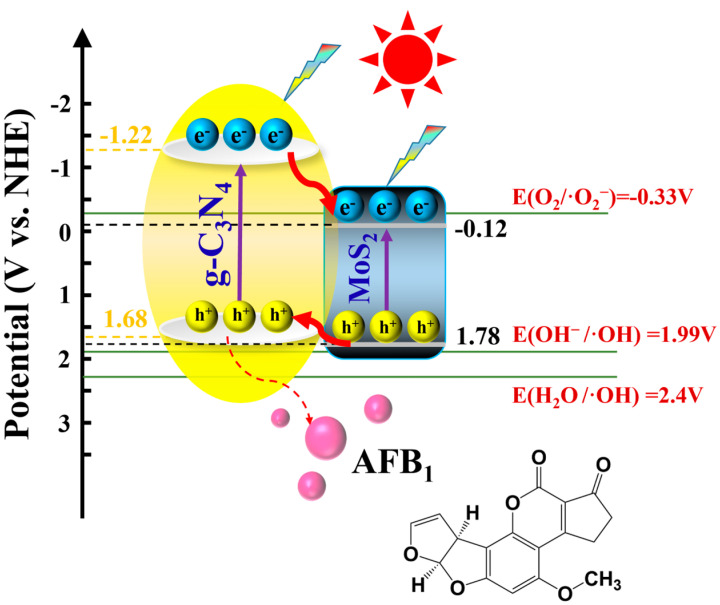
The photocatalytic mechanism of PAN-g-C_3_N_4_/MoS_2_ electrospun membranes for degradation of AFB_1_ [53].

Based on the previous results, the possible photocatalytic mechanism of AFB_1_ degradation by the PAN-g-C_3_N_4_/MoS_2_ electrospun membranes was proposed (Figure 10). It could be regarded that g-C_3_N_4_/MoS_2_ anchored on PAN electrospun membranes was simultaneously excited under visible light irradiation and produced photo-induced electrons and holes. According to previous studies and band gap values estimated by the Kubelka–Munk formula, the conduction band of g-C_3_N_4_ (−1.22 eV) is higher than that of MoS_2_ (−0.12 eV), and the valence band of MoS_2_ (1.78 eV) is lower than that of g-C_3_N_4_ (1.68 eV) [53]. The photo-induced electrons produced in g-C_3_N_4_ can be easily transferred to the conduction band of MoS_2_ through the interface, and the photo-induced holes produced in MoS_2_ transfer to the valence band of g-C_3_N_4_ in a similar manner. As a result, the photo-induced electrons are gathered in the conduction band of MoS_2_, and the photo-induced holes are gathered in the valence band of g-C_3_N_4_, which leads to photo-induced electrons and holes to separate effectively. Therefore, the probability of photo-induced electron-hole recombination is hindered, and the photocatalytic efficiency is improved accordingly. However, the conduction band potential of MoS_2_ is more positive than the potential of E(O_2_/•O_2_^−^) (−0.12 V > −0.33 V) [54]; the electrons on the conduction band of MoS_2_ cannot react with O_2_ to generate •O_2_^−^. For the same reason, the holes on the valence band of g-C_3_N_4_ cannot generate •OH, as the valence band of g-C_3_N_4_ is more negative than the potential of E(OH^−^/•OH) or E(H_2_O/•OH) (1.56 V < 1.99 or 2.4 V) [55]. Thereby, rich holes in the valence band of g-C_3_N_4_ act as the main reactive species to oxidize AFB_1_ directly, consistent with the results of active species trapping experiments. The reaction formulas are as follows:

g-C_3_N_4_/MoS_2_ + hν → e^−^(CB) + h^+^(VB) (2)

AFB_1_ + h^+^ → CO_2_ + H_2_O + intermediate products (3)

## 3. Conclusions

Three kinds of flexible electrospun membranes anchored with g-C_3_N_4_/MoS_2_ composites were synthesized via uniaxial or coaxial electrospinning techniques. Due to more g-C_3_N_4_/MoS_2_ photocatalysts being exposed and more active sites being produced, the photocatalytic efficiency of S_1_, S_2_, and S_3_ increased gradually. The degradation efficiency of AFB_1_ solution with a concentration of 500 PPb (50 mL) was up to 97% in 60 min under visible light irradiation with 0.025 g S_3_. The mechanism of photocatalytic membranes degradation of AFB_1_ in the photocatalytic process was proposed based on active species trapping experiments, and the reusability and stable activity were confirmed after five cycles of photocatalytic degradation experiments. Thus, the PAN-g-C_3_N_4_/MoS_2_ electrospun membranes were proved as high photocatalytic activity, easy separation, good reusability, and potential practical application in the foodstuff for the degradation of AFB_1_.

## 4. Materials and Methods

### 4.1. Materials and Reagents

AFB_1_ was purchased from Beijing Puhuashi Technology Development Co., Ltd. (Beijing, China), and dissolved to a certain concentration with deionized water. Melamine (≥99.0% purity), sodium molybdate (≥99.0% purity), thioacetamide (≥99.0% purity), N,N-dimethylformamide (DMF, AR, 99.5%), N-methyl pyrrolidone (NMP, ≥99.0%), anhydrous ethanol (AR, 99.5%), glacial acetic acid (for HPLC, ≥99.9%), trifluoroacetate (for HPLC, ≥99.5%), methanol (for HPLC, ≥99.9%), and acetonitrile (for HPLC, ≥99.9%) were purchased from Macklin Biochemical Co., Ltd. PAN (Mw ≈ 120,000) and PEO (Mw ≈ 200,000) were obtained from Sigma-Aldrich Co., Ltd. All reagents were used without any further purification. The deionized water used in this study was purified by a Millipore system.

### 4.2. Preparation of g-C_3_N_4_/MoS_2_

**Scheme 1:**
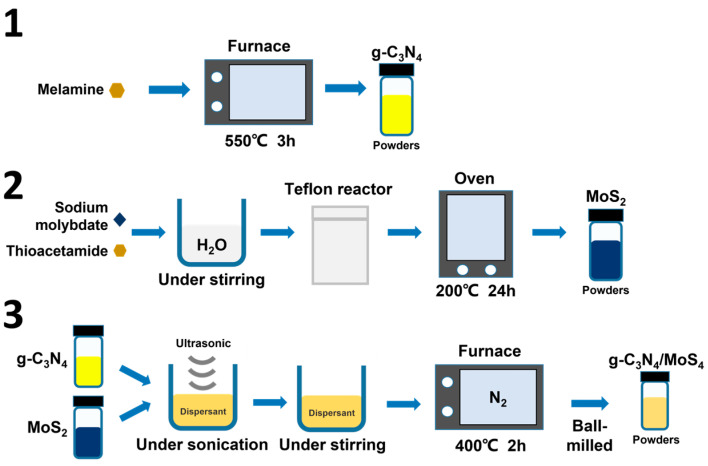
The schematic illustration of the fabrication of g-C_3_N_4_, MoS_2_, and g-C_3_N_4_/MoS_2_.

As shown in Scheme 1, the g-C_3_N_4_ powders were prepared by calcining melamine at 550 °C for 3 h (5 °C/min). The MoS_2_ powders were prepared by hydrothermal process. In a typical procedure, 20 mg sodium molybdate and 25 mg thioacetamide were dissolved in 30 mL deionized water under magnetic stirring for 20 min. Then, the above solution was poured into a stainless-steel autoclave, and the reaction temperature was controlled at 200 °C by the oven for 24 h. Following several times washing with deionized water and ethanol, the resultants dried at 60 °C for 10 h under vacuum were MoS_2_ powders.

The g-C_3_N_4_/MoS_2_ composites were fabricated by low-temperature calcination, and the mass ratio of MoS_2_ in g-C_3_N_4_/MoS_2_ was determined as 1% in this study. Firstly, 198 mg g-C_3_N_4_ and 2 mg MoS_2_ powders were dispersed in NMP and absolute ethanol, respectively, and ultrasonicated for 60 min. The two solutions were then mixed and stirred for 12 h, and the precipitates obtained after centrifugation were washed with deionized water and ethanol several times and dried at 80 °C for 10 h under vacuum. Secondly, the precipitates were ground to powders and followed by annealing at 400 °C for 2 h with a ramping speed of 5 °C/min in a nitrogen atmosphere. Finally, the g-C_3_N_4_/MoS_2_ composites were ball-milled for 3 h after cooling to room temperature for future use. According to the above scheme, the g-C_3_N_4_/MoS_2_ composites with different MoS_2_ mass contents 0.5%, 1.5%, 2%, and 2.5% were prepared by changing the amount of MoS_2_ added.

### 4.3. Preparation of PAN-g-C3N4/MoS2 Electrospun Membranes

**Scheme 2:**
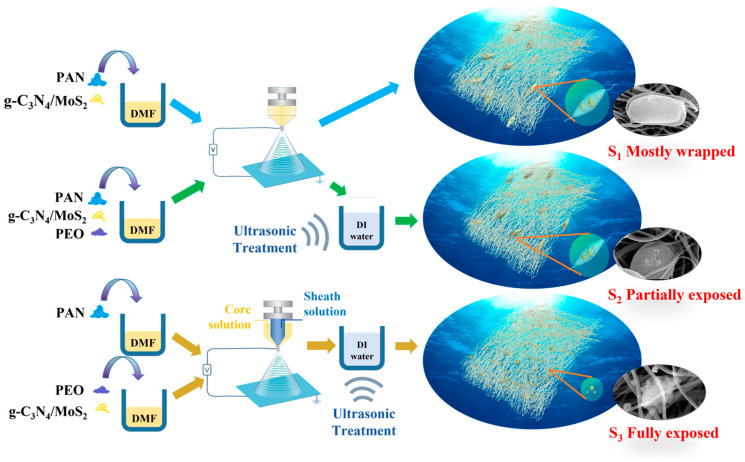
The schematic illustration of the fabrication of S_1_, S_2_, and S_3_.

Three kinds of PAN-g-C_3_N_4_/MoS_2_ electrospun membranes were fabricated by electrospinning (Scheme 2). For the first one, a certain amount of g-C_3_N_4_/MoS_2_ composites was added into DMF and ultrasonicated for 1 h to disperse the photocatalysts. Subsequently, PAN was added and stirred for 2 h to obtain a yellow-grey solution. The concentration of PAN in DMF was 12 *w*/*v*%, and the contents of g-C_3_N_4_/MoS_2_ composites to DMF was 3 *w*/*v*%. The prepared solution was then injected into a plastic syringe with a metal needle driven by a syringe pump at a flow rate of 1.5 mL/h for electrospinning. The applied voltage was 10 kV, and the distance from the metallic needle to the aluminum foil surface was 15 cm. After electrospinning, the electrospun membranes were dried at 60 °C under vacuum for 12 h, recorded as S_1_. The second one was prepared according to S_1_ with some modifications. Typically, the polymer added into DMF was changed to PAN/PEO (PAN: PEO = 2:1, wt%), while keeping the total concentration of the polymer constant with S_1_ (12 *w*/*v*%). After drying at 60 °C under vacuum for 12 h, the electrospun membranes were immersed in deionized water, sonicated in a water bath for 1 h, and placed at 60 °C for 24 h to fully wash out PEO. The washed electrospun membranes were dried at 60 °C under vacuum for 12 h, recorded as S_2_. The third one was prepared by a simple coaxial electrospinning technique. The core solution with concentration PAN 12 *w*/*v*% was prepared similarly to S_1_ without adding g-C_3_N_4_/MoS_2_ composites. The sheath solution was prepared with PEO, g-C_3_N_4_/MoS_2_, and DMF similar to S_1_. The concentration of PEO in DMF was set to 7 *w*/*v*%, and the contents of g-C_3_N_4_/MoS_2_ composites to DMF were 3 *w*/*v*%. The core and sheath solution was pumped out at rates of 1.5 mL/h using two syringe pumps, and the applied voltage and the distance from the metallic needle to the aluminum foil surface were set to be the same as both S_1_ and S_2_. The resultant electrospun membranes were washed with deionized water and dried at 60 °C under vacuum for 12 h, recorded as S_3_.

### 4.4. Characterization of PAN-g-C_3_N_4_/MoS_2_ Electrospun Membranes

The morphologies of the PAN-g-C_3_N_4_/MoS_2_ electrospun membranes were observed by SEM (ZEISS Sigma, Aalen, Germany), and the microstructure of g-C_3_N_4_/MoS_2_ composites were observed by TEM (JEM-2100F). XRD patterns was obtained with an X-ray diffractometer (MiniFlex 600, Tokyo, Japan) at a scanning speed of 2°/min. FTIR spectra were analyzed on a Vector-22 spectrometer. High-resolution XPS spectra were analyzed by an X-ray photoelectron spectrometer. DRS was detected by a UV/VIS spectrophotometer (Shimadzu UV-3600 Plus, Tokyo, Japan). TPC curves were tested on a three-electrode electrochemical workstation (CHI600E, Beijing, China) with PAN-g-C_3_N_4_/MoS_2_ electrospun membranes/glassy as the working electrode, Ag/AgCl as the reference electrode, and platinum wire as the counter electrode, respectively. The electrolyte was 0.1 M Na_2_SO_4_ aqueous solution.

### 4.5. Photocatalytic Degradation Experiment

The degradation of AFB_1_ was evaluated in an aqueous medium under visible light irradiation by a 300 W xenon lamp with a 400 nm cut-off filter. Samples from electrospun membranes were cut into a circular shape (2 cm in diameter and approximately 0.025 g in weight) and fixed on a bracket, immersed in 50 mL of AFB_1_ aqueous solution (500 PPb). Then, it was placed in the dark for 30 min to establish the adsorption/desorption equilibrium before light irradiation. The distance between the xenon lamp and the aqueous surface was 10 cm. In the progress of the photocatalytic degradation, 0.5 mL of the AFB_1_ aqueous solution was collected every 10 min and then added 0.25 mL glacial acetic acid and 0.25 mL trifluoroacetic acid. The mixed solution was put in a water bath at 70 °C for 40 min to enhance the fluorescence emission intensity of AFB_1_ when detected by HPLC. The concentration of the AFB_1_ was analyzed by the HPLC on Waters-600 equipped with a UV/Vis detector (emission wavelength at 365 nm) and C-18 Phenomenex reverse phase column (250 × 4.6 mm i.d., 5 μm) at a flow rate of 1 mL/min with an isocratic system composed of water: methanol: acetonitrile (70:20:10). Different factors were also analyzed, such as pH values (4–10) and initial concentration of AFB_1_. The AFB_1_ solution without electrospun membranes upon irradiation was also monitored in order to quantify the photocatalytic degradation of AFB_1_. The stability of the electrospun membranes was evaluated over 5 continuous cycle experiments under visible light irradiation. After each cycle, the electrospun membranes were rinsed with deionized water for continued use.

To explore the mechanism of degradation of AFB_1_ by the electrospun membranes, active species trapping experiments were carried out by using the addition of IPA (1 mM), AO (1 mM), and BQ (1 mM) to capture hydroxyl radicals (•OH), photogenerated holes (h^+^), and super-oxide anion radicals (•O_2_^−^), respectively.

## Figures and Tables

**Table 1:** Studies have reported the photocatalytic detoxification of mycotoxin.

Pollutant (Concentration)	Medium	Catalyst	Source	Time	Degradation	Ref (Year)
AFB_1_ (0.5 µg/mL)	Aqueous	g-C_3_N_4_ (0.1 mg/mL)	Xenon lamp (300 W, λ ≥ 400 nm)	120 min	70.20%	[23] Mao et al. (2018)
AFB_1_ (0.54 µg/mL)	Aqueous	WO_3_/RGO /g-C_3_N_4_ (0.1 mg/mL)	Xenon lamp (300 W, λ ≥ 420 nm)	120 min	92.40%	[25] Mao et al. (2018)
AFB_1_ (0.426 µg/mL)	Aqueous	WO_3_/CdS	Visible light irradiation (λ ≥ 420 nm)	80 min	95.50%	[22] Mao et al. (2019)
AFB_1_ (0.5~2 µg/mL)	Methanol	AC/TiO_2_ (0.3 mg/mL)	Mercury lamp (130 W, 350–450 nm)	120 min	98%	[26] Sun et al. (2019)
AFB_1_ (0.5 µg/mL)	Aqueous	TiO_2_ /UiO-67 (0.1 mg/mL)	Xenon lamp (300 W, λ ≥ 420 nm)	80 min	98.90%	[27] Zhang et al. (2022)
AFB_1_ (0.5~30 μg/mL)	Aqueous/Soymilk	ZnO, Fe_2_O_3_, MnO_2_ and CuO (0.1 mg/mL)	UV irradiation	60 min	±95%	[28] Raesi et al. (2022)
AFB_1_/AFB_2_/ AFG_1_/AFG_2_ (315.21 µg/kg)	Peanuts	g-C_3_N_4_ /NiFe_2_O_4_ (2 mg/mL)	Xenon lamp (300 W, λ ≥ 420 nm)	90 min	94.10%	[29] Sun et al. (2021)
DONs (15 µg/mL)	Aqueous	Graphene /ZnO (0.5 mg/mL)	UV irradiation	120 min	99.00%	[20] Sun et al. (2017)
DONs (4 µg/mL)	Aqueous	α-Fe_2_O_3_ (0.1 mg/mL)	Xenon lamp (300 W, λ ≥ 420 nm)	120 min	90.30%	[30] Mao et al. (2019)

## Data Availability

Raw data are available on request.

## Supplementary Materials

The following supporting information can be downloaded at: https://www.mdpi.com/article/10.3390/toxins15020133/s1, Figure S1: Photocatalytic degradation of RhB with different weight ratios of g-C3N4 and MoS2.
