# Supplementary material for: Electrospun Membranes Anchored with g-C3N4/MoS2 for Highly Efficient Photocatalytic Degradation of Aflatoxin B1 under Visible Light
Source: Toxins (Basel). 2023 Feb 6;15(2):133. doi: 10.3390/toxins15020133 (PMC9960316; doi:10.3390/toxins15020133)
Supplement: Supplementary file 1 [file toxins-15-00133-s001.zip › toxins-2199551-supplementary.pdf]

Supplementary Information: Electrospun Membranes Anchored with g-C_3N_4/MoS_2 for Highly Efficient Photocatalytic Degradation of Aflatoxin B_1 under Visible Light

1. Computational Methods

According to the X-ray diffraction theory, when the crystallite size is less than 100 nm, the diffraction peak width becomes significant as the crystallite size decreases. Considering the absorption effect of the sample and the influence of the structure on the diffraction pattern, the crystallite size of the sample can be calculated by Debye-Scherrer Formula (S1):

$$FW(S)^{D} = FWHM^{D} - FW(I)^{D}$$

$$Size = \frac{K\lambda}{FW(S)\cos(\theta)} \tag{S1}$$

where *FW(S)*, *FWHM*, *FW(I)*, λ and θ are the broadening of diffraction peak, the full width at half maximum of diffraction peak, the full width of diffraction peak, X-ray wavelength and diffraction angle, respectively. The values of *K* and *D* are constants, and the value of *D* is determined by the diffraction peak shape. Additionally, *Size* usually represents the length of a specific diffraction plane direction.

2.Characterization results

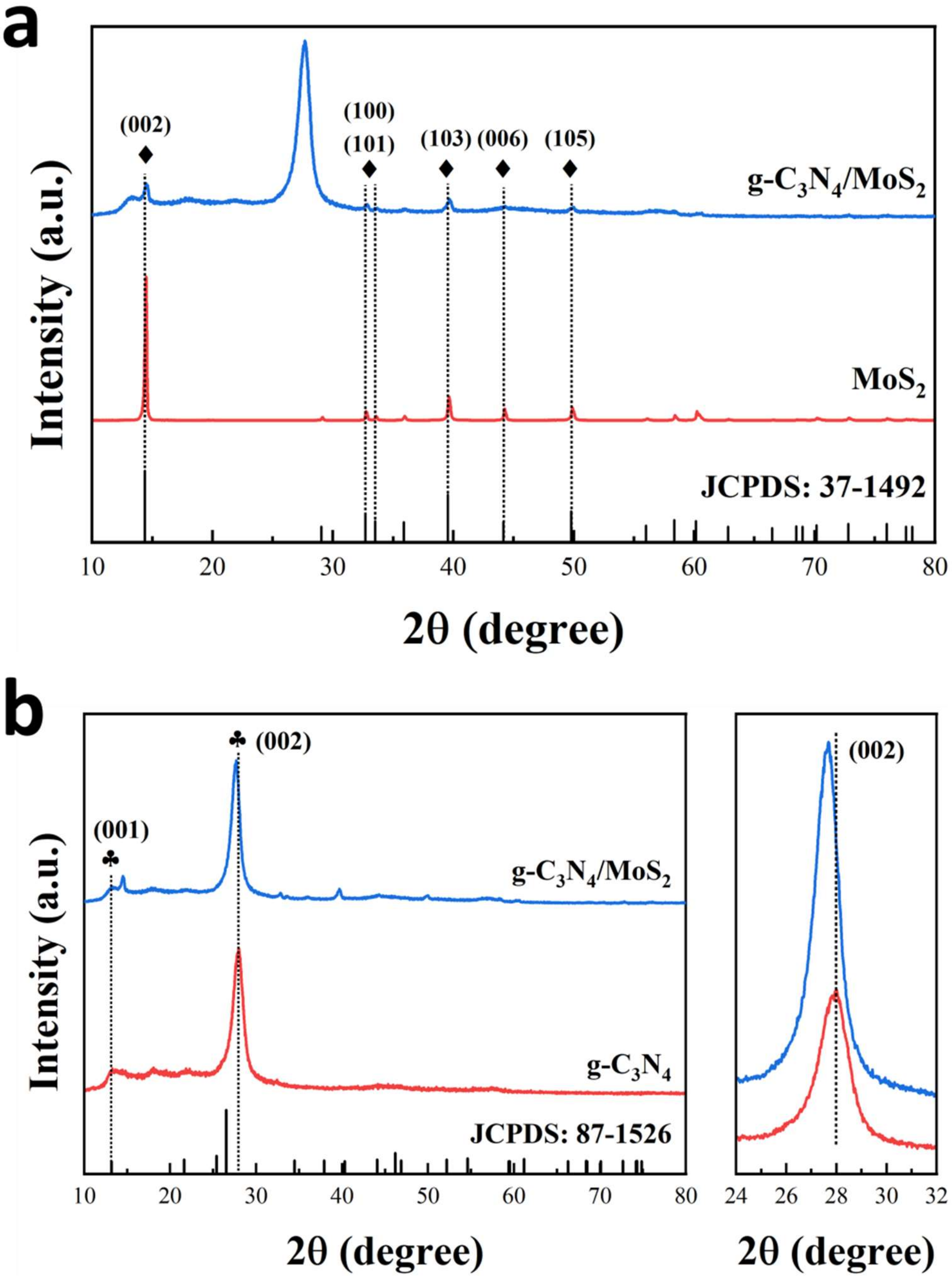

Figure S1. XRD patterns of g-C_3N_4/MoS_2 compared with (a) MoS_2 and (b) g-C_3N_4.
